# Decomposition of SO_2_ on Ni(111) Surface and the Effect of Metal Doping: A First-Principles Study

**DOI:** 10.3390/molecules28186739

**Published:** 2023-09-21

**Authors:** Lingtao Liu, Chenxin Zhang, Wenshou Wang, Genghong Li, Bingtian Zhu

**Affiliations:** SINOPEC Research Institute of Petroleum Processing Co., Ltd., Beijing 100083, China

**Keywords:** SO_2_, adsorption, decomposition, Ni(111), doping, DFT

## Abstract

Sulfides poisoning of metallic Ni is an important issue in catalyst deactivation. SO_2_, similar to H_2_S and other sulfides, is an impurity presented in reactants or during the regeneration steps. Herein, spin-polarized density functional theory calculations were used to study the adsorption and decomposition of SO_2_ on a pristine and metal-doped Ni(111) surface. The adsorption energy, transition state energy, and partial density of state (PDOS) were calculated. On the pristine Ni(111) surface, ten different configurations were considered, and three typical ones were selected for transition state searching. It was found that the reaction barrier of the first S-O bond dissociation was much higher than that of the second one. Doping the top layer with a second metal could strongly change the adsorption and decomposition behavior. Doping with 3/9ML Co slightly increases the adsorption energy of SO_2_ for most configurations and decreases the reaction barriers of the SO_2_-*tht*-2 decomposition, while the others decrease the adsorption ability and increase the barriers. The order of adsorption energy for the most stable configurations is Co > Ni > Cu > Rh > Pd. The order of the first S-O bond dissociation reaction barriers is Pd > Rh > Cu = Ni > Co, and the order of the second bond dissociation barrier is Rh > Pd > Cu > Ni > Co.

## 1. Introduction

SO_2_ is an important raw material that produces bulk chemicals, and it is also a major air pollutant, which kills lives [1], erodes metals [2,3,4], and poisons catalysts [5,6,7]. The interaction of SO_2_ with metal is drawing researchers’ attention because it plays an important role in the environment and industry [8].

One of the main sources of SO_2_ is the burning of fossil fuels. Desulfurization is a widely used strategy to decrease SO_2_ emissions from fossil fuels [9,10]. The S Zorb process of SINOPEC Corp., as a typical reactive adsorption desulfurization (RADS), shows its distinguished advantages [11,12,13,14,15]. It is carried out in a circulating fluidized bed reactor using a sorbent with Ni and ZnO as the main active phases. In the reactor, sulfur is transferred to the sorbent under a H_2_ atmosphere to form ZnS. The sorbent with a particular sulfur loading is then regenerated using air to convert ZnS back to ZnO with SO_2_. Although numerous efforts on revealing the chemistry of desulfurization reaction have been made [16,17,18], the behavior of nickel in the SO_2_-rich gas during regeneration is still unclear. A deep understanding of the SO_2_ reaction on metal surfaces would provide insights that would help develop more efficient catalysts and processes.

Despite the sulfur-removing strategies, SO_2_ still remains a question for catalyst scientists. Using density functional theory (DFT), Benjamin D. Gould et al. studied the effect of different sulfides on the Pt of a fuel cell electrode and found that SO_2_ had a poisoning ability similar to H_2_S and COS [19]. This is due to the strong adsorption energy of S on Pt’s surface [20,21], which is a similar case for sulfides deactivating Ni [21]. The three-way automotive catalysts could also be poisoned by SO_2_ in the tail gas [22].

Based on those issues, understanding SO_2_ adsorption and conversion over a metal surface could shed light on developing new catalysts. A few groups have devoted themselves to studying the adsorption of SO_2_ on different transition metal surfaces using first-principles calculations, such as Ni [23,24,25], Cu [26,27], Pt [28,29,30,31], and others [32]. Xi Lin reported 20 different configurations of SO_2_ on the Pt (111) surface and figured out the most energetically stable configurations [28]. Markus Happel et al. performed a combined experiment and density functional study on the adsorption of SO_2_ on clean and oxygen-precovered Pt(111), and also found that there were parallel or perpendicular geometries [30]. M. J. Ungerer et al. compared the interaction of SO_2_ with the Pt’s different surfaces and concluded that the order of adsorption energy was (001) > (011) > (111) [31]. Yoshiko Sakai et al. used cluster models with 4 and 15 Ni atoms and found that the most stable configuration was a molecular plane nearly parallel to the surface, with all the S and O atoms on bridge sites [23]. M.J. Harrison et al. used a slab model and concluded that the parallel–hollow geometry was the more preferred adsorption site on Ni [24]. Xin Wei et al. noticed that the existence of atomic O strengthened the adsorption capacity of SO_2_ on a pristine Ni(111) surface [25].

The conversion of SO_2_ was also studied. Chen-Hao Yeh studied the oxidation of S on Pt(111), Ni(111), and the core–shell structures [33]. They considered four absorbed structures and found that S could be oxidized with either atomic O or O_2_ on a Ni surface to form SO, continuing to be oxidized to SO_2_ of the parallel bridge orientation. The core–shell structure could change both the adsorption energy and the reaction barrier for SO_2_ formation. H. N. Sharma et al. used DFT calculations coupled with microkinetic modeling to examine the oxidation of SO_x_ to SO_4_ on Pd(111) and Pt(111) surfaces, in which three different orientations were considered [34]. Natasha M. Galea studied removing sulfur from Ni by O_2_ using a *p*(2 × 2) three-layer model and found that atomic sulfur could be removed up to an initial coverage of 50% at high temperatures [35].

In this study, we used DFT to investigate the adsorption of SO_2_ on the Ni(111) surface with different configurations and studied the decomposition pathways of the three most stable ones. The effect of doping metals, namely Cu/Co/Pd/Rh, on whether a second metal would improve the tolerance of SO_2_ relative to pure Ni was further modeled.

## 2. Results

### 2.1. Absorption of S and O on Different Metal Surfaces

**Figure 1:**
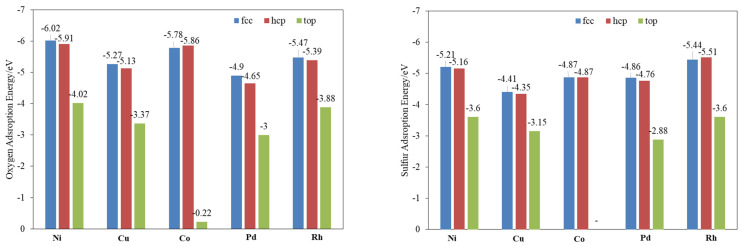
Binding energies of O and S on different metal (111) surfaces.

Understanding the nature of sulfur and oxygen bonding with different metals is decisive for investigating the decomposition of SO_2_. The adsorption energy (Figure 1) and bond length (Figure S1) of O and S on three typical sites of different metal surfaces were calculated. The results show that the sulfur and oxygen over these metals’ surfaces are strongly chemisorbed. The Fcc site is the most stable for both atoms adsorbed on all the calculated metals except for S on Rh(111) and O on Co(111), of which the hcp site is more stable, while the top site has the weakest bind energy. For the adsorption energy of O, Ni shows the highest value, and rest are in the following order: Co > Rh > Cu > Pd. S is a little different from O, the adsorption of which is in the order of Rh > Ni > Pd > Cu > Co.

### 2.2. SO_2_ on Ni(111)

#### 2.2.1. Configurations of SO_2_ on Ni(111) Surface

**Figure 2:**
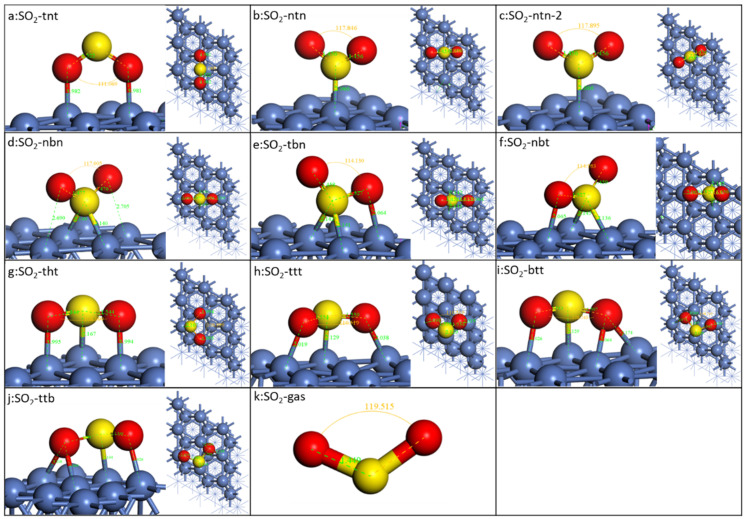
Optimized geometries of SO_2_ on Ni(111) surface. The blue, red, and yellow spheres represent Ni, O, and S atoms, respectively.

The binding energetic and geometric data of the stable configurations are presented in Figure 2 and Table 1. The S-O bond length of gaseous SO_2_ is 1.449 Å, and the bond angle of ∠O-S-O is 119.5°. There are ten different configurations identified, as shown in Figure 2, which could be categorized into four groups: (1) binding with two O on the Ni top site and the molecular plane perpendicular to the Ni surface (a: SO_2_-*tnt*); (2) binding with only S and the molecular plane perpendicular to the Ni surface (b: SO_2_-*ntn*/c: SO_2_-*ntn*-2/d: SO_2_-*nbn*). SO_2_-*ntn* and SO_2_-*ntn*-2’s S are on Ni’s top site while SO_2_-*nbn*’s is on the bridge site; (3) S on the bridge site, and one O on the top site (e: SO_2_-*tbn*/ f: SO_2_-*nbt*); (4) all atoms of SO_2_ binding to the metal surfaces and the molecular plane parallel to the metal surface (g: SO_2_-*tht*/h: SO_2_-*ttt*/i: SO_2_-*btt*/j: SO_2_-*ttb*). The difference between SO_2_-*tht*) and SO_2_-*ttt*) is that two O atoms of the SO_2_-*tht*) are on the top site of two adjacent Ni atoms and those of SO_2_-*ttt*) are on the top site of two Ni surface, which are on the long diagonal of the equilateral diamond composed of four nickel atoms.

It can be found In Table 1 that when adsorbed on the Ni(111) surface, both the bond length and the angle of SO_2_ decrease. For all the configurations, SO_2_-*tnt* shows the lowest binding energy, even lower than SO_2_-*ntn* and SO_2_-*ntn*-2, although O has a higher adsorption energy than S on the top site of Ni. SO_2_-*ntn*-2 is the rotation about the S-metal bond of SO_2_-*ntn*, which has a small effect on the energy, meaning that SO_2_ of this type could rotate almost freely on the surface. SO_2_-*nbn* has a much higher adsorption energy than SO_2_-*ntn* and SO_2_-*ntn*-2 because its S is on the bridge site. SO_2_-*tbn* and SO_2_-*nbt* are quite similar with S on the bridge site and O on the top site. They show the highest binding energy, −1.22 eV and −1.23 eV, respectively, which might be due to the fact that S on the bridge site is much more stable than the top site. A higher result of −1.40 eV is obtained using a different function [24]. The similar configuration of SO_2_ on Pt(111) is also the most stable SO_2_ on Pt(111) [28,31] and Ir(111) [32]. SO_2_-*tht*’s binding energy is higher than SO_2_-*ttt*, SO_2_-*btt*, and SO_2_-*ttb*. The same configuration as the reported was also identified with a slightly higher adsorption energy (−1.11 eV vs. −1.06 eV) [33].

The length of the S-O bond of all adsorbed SO_2_ is longer than the gaseous SO_2_, indicating that adsorption could stretch the bond, which would decrease the bond energy. The angles are smaller than the gas phase, decreasing to the range of between 109.0° and 117.8°. The smallest angle is SO_2_-*btt*, which is caused by two O binding to two adjacent nickel atoms. However, there is no simple numerical relationship between the binding energy and the bond length or angle.

#### 2.2.2. SO_2_ Decomposition on Ni(111)

As types SO_2_-*tbn*, SO_2_-*nbt*, and SO_2_-*tht* have the highest binding energy, they would be the dominant configurations on the surface. To obtain the difference of decomposition pathways, we chose SO_2_-*tbn*, SO_2_-*tht*, and SO_2_-*ttt* to study the decomposition behavior of SO_2_ on the pristine Ni surface.

**Pathway1: SO_2_-*tbn***

**Figure 3:**
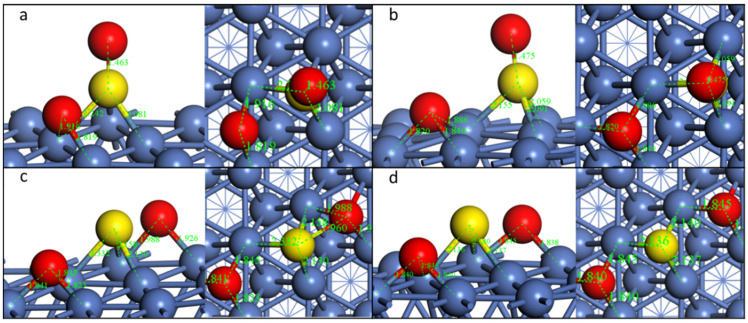
Geometric structures of the dissociation of SO_2_-*b-4* on Ni(111) surface. ((**a**,**b**): TS and FS of the first S-O dissociation; (**b**–**d**)): IS, TS, and FS of the second S-O dissociation).

When searching for the TS, IS as well as FS must be recognized beforehand. For the dissociation of the first S-O bond, the final state is shown in Figure 3b. The dissociated O is on the fcc site, while the left SO group is vertical to the nickel surface, and the S is on the nearby fcc site. This step is energetically favorable, with an energy change of −0.44 eV. During the first S-O dissociation, O on the top site moves to the nearest nearby fcc site, and the left SO group moves to the fcc site with the S-O bond vertical to the Ni(111) surface. The distance of S and O increases from 1.459 Å to 2.975 Å, indicating the breakdown of this S-O bond (Table 2). However, the left S-O bond length decreases from 1.527 Å to 1.475 Å. The transition state is similar to the final state. The reaction barrier of this step is 0.79 eV.

For the dissociation of the second S-O bond, the FS is shown in Figure 3c. The second detached O is also on the fcc site, while the left S is on its original site. The second S-O dissociation takes place when the O moves to the fcc site and the distance increases from 1.475 Å to 2.579 Å, meaning a total decomposition of SO_2_ to S and O adsorbed on Ni(111). This step is almost energetic neutral, while the reaction barrier is 0.39 eV, which is much lower than the first step. The overall decomposition is exothermic.

**Pathway2: SO_2_-*tht***

**Figure 4:**
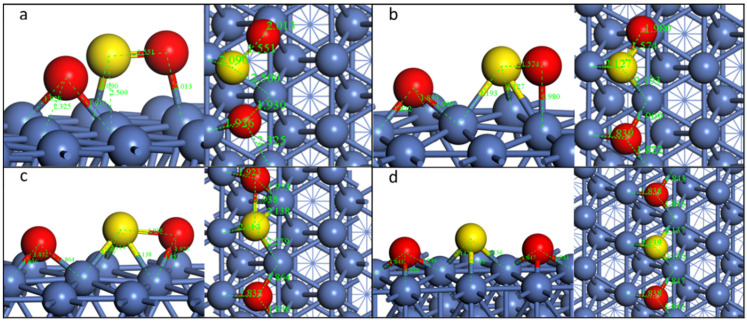
Geometric structures of the dissociation of SO_2_-*tht* on Ni(111) surface. ((**a**,**b**): TS and FS of the first S-O dissociation; (**b**–**d**)): IS, TS, and FS of the second S-O dissociation).

For the decomposition of SO_2_-*tht*, the FS of the two steps is shown in Figure 4. Similar to the Pathway1, for the first S-O dissociation, the O on the top site moves to the nearby fcc site. Meanwhile, the S of the left SO group migrates to the bridge site, while the O is almost unmoved. The S-O bond is nearly parallel to the Ni(111) surface. The distance of S and O increases from 1.544 Å to 2.758 Å (Table 3), indicating the breakdown of this S-O bond. For the other S-O bond, it increases from 1.544 Å to 1.574 Å, which shows a different tendency from Pathway1. This is because for Pathway2, both atoms of SO bind to the Ni surface, stretching the S-O bond.

The second S-O dissociation takes place when the O moves from the top site to the nearby fcc site and the distance increases from 1.574 Å to 2.573 Å. The reaction barriers of the two steps for this configuration are smaller than the first path, especially for the second step, which decreases from 0.39 eV to 0.17 eV.

**Pathway3: SO_2_-*ttt***

**Figure 5:**
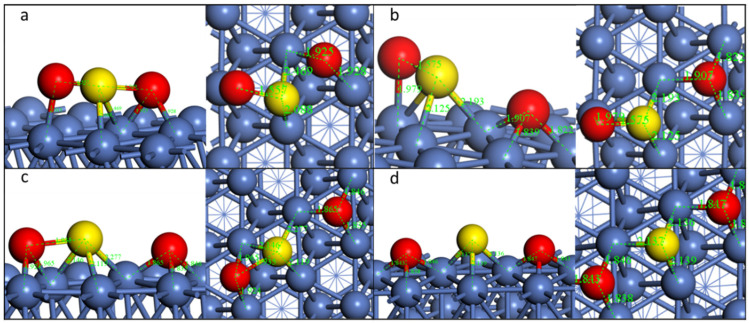
Geometric structures of the dissociation of SO_2_*-ttt* on Ni(111) surface. ((**a**,**b**): TS and FS of the first S-O dissociation; (**b**–**d**)): IS, TS, and FS of the second S-O dissociation).

The first S-O dissociation of Pathway3 is similar to Pathway2, and the second step of decomposition is almost the same (Figure 5). The reaction barriers are the lowest among the three pathways (Table 4).

**Figure 6:**
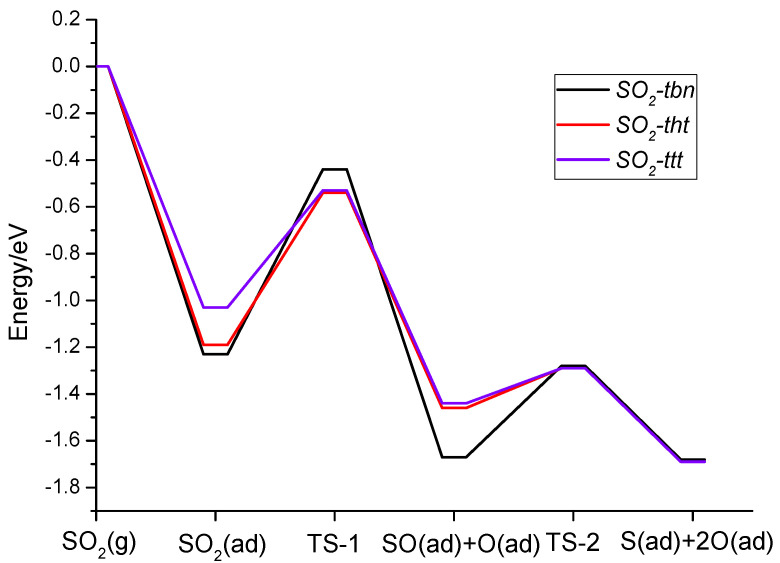
Calculated possible potential energy diagram of electronic energy for SO_2_ decomposition on Ni(111).

Figure 6 presents a detailed energy profile for the dissociation of the three different adsorbed SO_2_ configurations. It must be noted that for all three pathways, the overall decomposition processes are exothermic. The first step has a higher energy barrier compared with the second one. The higher barriers for the SO_2_-*tbn* type is attributed to the fact that the dissociated O does not bond with the nickel surface, which cannot decrease the bond energy as in the other two cases.

### 2.3. SO_2_ on Doped Ni(111)

#### 2.3.1. Doping Effect

Doping with a second element usually changes a catalyst’s property [36,37,38]. Nishith K. Das studied the effect of Cr doping on the adsorption of S, O, SO. and SO_2_ over the Ni(111) surface and found that Cr doping of the Ni(111) surface increased their adsorption energies [39]. Chen-Hao Yeh studied the core–shell structure of Ni@Pt and Pt@Ni and found that the Pt@Ni(111) surface exhibited less affinity for SO_x_ or S than pure Pt(111), while Ni@Pt showed the opposite results [33]. We herein studied the doping effect of Co/Cu/Rh/Pd on Ni(111). Three Ni atoms on the first layer were replaced by the doping metal. Three topologies were considered (Figure S3). The dispersion of Co and Cu had a minor effect on the energy, while the uniformly dispersed Rh or Pd showed the lowest energy. Thus, we chose the uniformly dispersed surfaces to study.

#### 2.3.2. Adsorption

**Figure 7:**
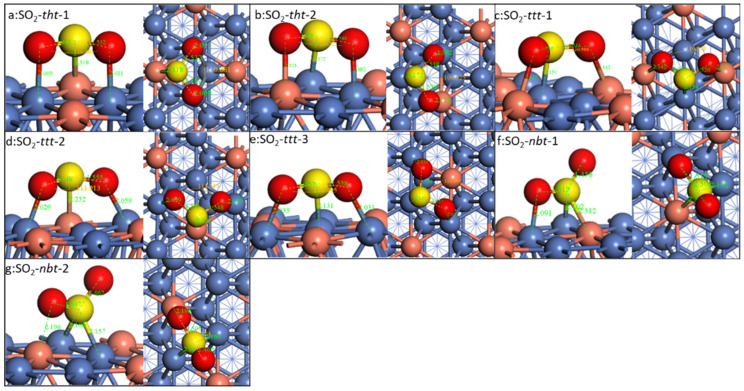
Configurations of SO_2_ on Cu-doped surface. The blue, red, yellow, and brass spheres represent Ni, O, S, and Cu atoms, respectively.

The adsorption of SO_2_ on different doped Ni(111) is compared. As there are too many different configurations if all the possibilities were considered, we chose two similar ones with the most stable configurations as on the pristine Ni(111) surface, namely SO_2_-*nbt,* SO_2_-*tht,* and *SO_2_-ttt*. On the doped Ni(111), there are seven different configurations. Taking Cu-doped Ni(111), for example (Figure 7), for the SO_2_-*tht* types, when S is on Cu’s top site, the two O atoms are on Ni’s top site. And when the S is on Ni’s top site, one O is on Ni’s top site and the other is on Cu’s top site. For the SO_2_-*nbt* and SO_2_-*ttt* types, there are three and two different configurations, respectively. This is similar for all other doped surfaces.

Table 5 shows the binding energies on different surfaces. The change in binding energies is quite different. On the Cu-doped Ni(111) surface, the SO_2_-*tht* and SO_2_*-bnt* types’ binding energies all decreased compared with the pristine Ni(111) surface. It must be noted that when S binds to Cu, the binding energy is weaker than the binding energy when O binds Cu. Thus, SO_2_-*tht*-1 has a lower binding energy than SO_2_-*tht*-2. *SO*_2_*-ttt*-3 has even higher energy than on the pristine Ni(111) surface (−1.09 eV vs. −1.03 eV). This thus concludes that *SO_2_-ttt*-3, SO_2_-*nbt*-2, and SO_2_-*tht*-2 would be the most abundant configurations on the Cu-doped Ni surface.

The Co-doped Ni surface is a bit different from the Cu-doped surface. The SO_2_-*tht*-1 has higher bind energy than that on the pristine Ni, while SO_2_-*tht*-2 is a little lower. For the *SO_2_-ttt* types, all the configurations have higher binding energy than that on the pristine Ni. SO_2_-*nbt*-2 shows the highest binding energy among all the configurations.

Rh and Pd doping have a similar trend. All the configurations have a much lower binding energy than that on the pristine Ni. Meanwhile, the most abundant configurations are SO_2_-*tht*-2 and SO_2_-*nbt*-2.

#### 2.3.3. Decomposition on Doped Metal Surfaces

**Figure 8:**
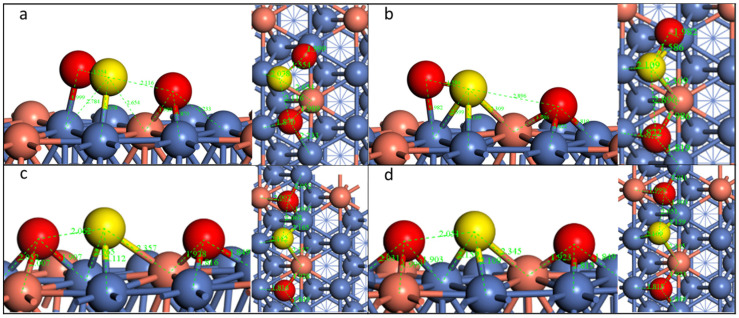
Geometric structures of the dissociation of SO_2_*-tht*-2 on Cu-doped Ni(111) surface. ((**a**,**b**): TS and FS of the first S-O dissociation; (**b**–**d**)): IS, TS, and FS of the second S-O dissociation.

For the doped Ni surface, we selected SO_2_-*tht*-2 to study the decomposition pathway. The transition state and final state are illustrated in Figure 8 and Figure S4.

On the Cu-, Co-, and Rh-doped surfaces, the reaction pathways are similar; thus, we still used Cu as the example for a detailed examination. When the first S-O bond dissociates, the O migrates to the nearby fcc site, and the S of the second SO moves to the fcc site underneath the previous S-O bond. However, the dissociated O is not in the center of the fcc site, leaning toward the bridge site of Ni-Ni, and the left SO deviates from Cu, not alike on the pristine Ni surface, which is along the median of the fcc site. This phenomenon originates from the fact that both O and S have a higher adsorption energy with Ni compared with Cu (Figure 1). The second S-O bond dissociates when the O moves to the nearby the fcc site. All S and O atoms are close to the fcc sites.

The configuration is a little different on the Pd-doped surface (Figure S5-3). Although the initial configuration is similar to that on the pristine Ni surface, when the first S-O bond dissociates, the leaving O migrates from the top site to the bridge site, and the left SO group lies across the Ni-Ni bond. When the second S-O dissociates, the S atom is on the bridge site at the transition state, and the O also moves to the bridge site. After the total dissociation of SO_2_, the first O is on the site nearby the fcc site, the S is on the bridge site, and the second O is on the hcp site. This result is due to the fact that Pd has a much lower affiliation ability with both O and S compared to Ni.

Although the adsorption energy also decreases for the same configuration on the Co-doped surface compared with the pristine surface as on other doped surfaces, the reaction energy for both dissociation steps increases and the reaction barriers all decrease (Table 6). The opposite phenomenon could be observed on other doped surfaces, among which the Pd-doped surface shows the highest reaction barriers for the determining step (first S-O dissociation) and becomes an endothermic process.

### 2.4. Analysis

**Figure 9:**
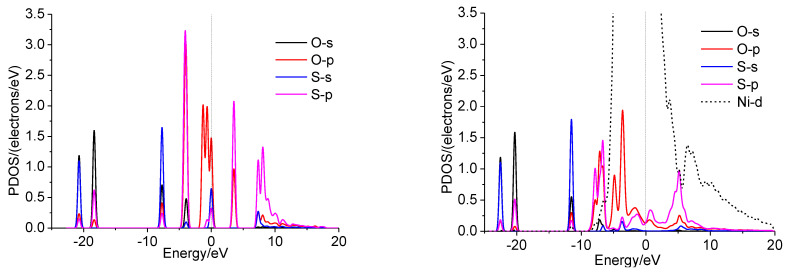
PDOS of SO_2_ (g) and SO_2_ adsorbed on Ni(111) surface.

The interactions of SO_2_ and different Ni surfaces were investigated in detail by the projected density of states (PDOSs). The results of Ni were chosen for the illustration (Figure 9). In the gas phase, the overlapping of the p orbit of the S and O atoms expresses the S-O bonds in SO_2_. For SO_2_, the interactions between the S and O atoms of the SO_2_ and Ni atoms of nickel surface can be explained by the p-DOS of S, O of SO_2_, and d-DOS of Ni. The redistribution of p-DOS of S and O indicates the bond formations of S and O atoms of SO_2_ with Ni. Similar cases are observed on doped surfaces (Figure S6).

## 3. Methods

Density functional theory (DFT) was performed on the CASTEP (Cambridge Sequential Total Energy Package) code implemented in Material Studio of Accelrys Inc., Cambridge, UK [40]. The electron exchange correlation energy was modeled with ultrasoft pseudopotentials and Perdew–Burke–Ernzerh (PBE) exchange–correlation functional based on the generalized gradient approximation (GGA). A wave function energy cutoff of 400 eV was used according to the literature [35,41]. A Fermi smearing of 0.1 eV was utilized. The spin polarization calculation was used when Ni was included in the model. The convergence criteria for the structure optimization and energy calculation were set to (a) an SCF tolerance of 1.0 × 10^−6^ eV/atom, (b) an energy tolerance of 1.0 × 10^−5^ eV/atom, (c) a maximum force tolerance of 0.03 eV/Å, and (d) a maximum displacement tolerance of 1.0 × 10^−3^/Å. For the adsorption of O and S on different metals, 2 × 2 supercell of a four-layer slab and the 6 × 6 × 1 k-point Monkhorst-Pack mesh were used. The SO_2_ adsorption, transition structures, and products of the reactions over the pristine and doped Ni(111) surface were modeled using periodic 3 × 3 supercell of a four-layer slab, and the 5 × 5 × 1 k-point Monkhorst-Pack mesh was used. For both cases, the slabs were separated with a vacuum spacing of 15 Å to minimize interactions between the slabs. All the metals have a face-centered cubic lattice. The calculated lattice parameters of the pure metals are 3.54 Å for bulk Ni for both 2 × 2 and 3 × 3 supercells, which are in satisfactory agreement with experimental observations of 3.52 Å. The lattice parameters of Co, Cu, Rh, and Pd were 3.46, 3.64, 3.89, and 3.93 Å, respectively. The corresponding experimental values were 3.54, 3.61, 3.80, and 3.89 Å, respectively. All of the calculated results were within 3% of the measured values. During geometry optimization, the bottom two layers were fixed, whereas the top two layers were allowed to relax.

The dissociation pathway was sequential abstraction of O atom from SO_2_. The transition states (TSs) were searched using the complete LST/QST method [42], and the convergence criterion of root-mean-square forces on atoms tolerance of was set to 0.05 eV/Å. The adsorption energy ΔE_ads,_ reaction energy (ΔE_r_), and activation barrier E_a_ was defined as follows:

ΔE_ads_ = E_A-S_ − (E_slab_ + E_adsorbate_)

ΔE_r_ = E_FS_ − E_IS_

E_a_ = E_TS_ − E_IS_

where E_A-S_ is the energy of the slab together with the adsorbate, and E_adsorbate_, E_slab,_ E_FS_, E_TS_, and E_IS_ are the total energy of the free adsorbate, bare slab, final state (FS), transition state (TS), and initial state (IS).

## 4. Conclusions

In summary, the periodic spin-polarized DFT calculation was exploited to gain a detailed understanding of the adsorption and decomposition of SO_2_ on pristine and metal-doped Ni(111) surfaces. The adsorption energy, reaction barrier, and partial density of state (PDOS) were calculated. On the pristine Ni surface, ten different configurations were considered, and three typical ones were selected to search for the transition states in order to reveal the reaction barriers. Among the three types, it was found that the barrier of the first S-O bond was much higher than the second one, and a lower reaction barrier was associated with weaker adsorption energy. Doping the top layer with a second metal could strongly change the adsorption and decomposition behavior. The order of adsorption energy for the most stable configurations is Co > Ni > Cu > Rh > Pd. The order of the first S-O bond dissociation reaction barriers is Pd > Rh > Cu = Ni > Co, and the order of the second bond dissociation barrier is Rh > Pd > Cu > Ni > Co. Overall, Pd is the best choice among the studied metals for decreasing the poisoning effect of Ni from SO_2_ because it decreases the adsorption energy of SO_2_ and increases the determining step of the decomposition reaction barriers the most.

## Figures and Tables

**Table 1:** Binding energies, bond lengths, and angles of SO_2_ on Ni(111).

	Configuration ^1^	ΔE_a_	Ni-S/Ni-O ^2^	S-O ^3^	∠O-S-O ^4^
a	SO_2_-*tnt*	−0.44	1.981/NM/1.982	(1.522, 1.522)	111.1
b	SO_2_-*ntn*	−0.65	NM/2.066/NM	(1.456, 1.456)	117.8
c	SO_2_-*ntn*-2	−0.66	NM/2.066/NM	(1.456, 1.456)	117.8
d	SO_2_-*nbn*	−1.08	NM/(2.140, 2.140)/NM	(1.476, 1.477)	117.0
e	SO_2_-*tbn*	−1.22	2.064/(2.141, 2.142)/NM	(1.458, 1.527)	114.1
f	SO_2_-*nbt*	−1.23	NM/(2.136, 2.145)/2.065	(1.459, 1.527)	114.2
g	SO_2_-*tht*	−1.19	2.167/(1.994, 2.734, 2.732)/1.995	(1.544, 1.544)	109.0
h	SO_2_-*ttt*	−1.03	2.129/2.019/2.038	(1.554, 1.559)	110.5
i	SO_2_-*btt*	−1.11	(2.064, 2.174)/2.129/2.026	(1.540, 1.597)	108.7
j	SO_2_-*ttb*	−1.09	2.100/2.138/(2.069, 2.074)	(1.543, 1.590)	108.8
k	SO_2_-*gas*	-	-	(1.449, 1.449)	119.5

^1^: *nbt*: *n*-one O atom is not bonded with Ni, *b*-S on the bridge site, *t*-the other O is on the top site; *h* refers to hollow site; ^2^: NM/α/(β, γ, δ):NM means not-measured; α~δ are the distances of O-Ni or S-Ni; (β, γ, δ) means O is on the hollow site, while (β, γ) means it’s on bridge site. All of the units are Å; ^3^: the bond length of two S-O; ^4^: units is °.

**Table 2:** Bond lengths and energy change in IS, TS, and FS for the dissociation of SO_2_-*tbn* on Ni(111) surface.

Configuration	Ni-O_a_	Ni-O_b_	Ni-S	S-O_a_	S-O_b_	ΔE_r_*
SO_2_-*tbn*	2.065	NM	2.136, 2.145	1.459	1.527	−1.23
TS-*tbn*-1	1.819, 1.916	NM	2.081, 2.145, 2.242	2.318	1.463	−0.44
SO-O-*tbn*	1.820, 1.840, 1.886	NM	2.059, 2.091, 2.155	2.975	1.475	−1.67
TS-*tbn*-2	1.823, 1.841, 1.845	1.926, 1.988	2.130, 2.158, 2.332	2.897	1.960	−1.28
S-O-O-*tbn*	1.840, 1.840, 1.845	1.838, 1.842, 1.845	2.136, 2.137, 2.140	2.574	2.579	−1.68

ΔE_r_*: compared to gaseous SO_2_ and bare Ni(111).

**Table 3:** Bond lengths and energy change of the IS, TS, and FS for the dissociation of SO_2_-*tht* on Ni(111) surface.

Configuration	Ni-O_a_	Ni-O_b_	Ni-S	S-O_a_	S-O_b_	ΔE_r_
SO_2_-*tht*	1.994	1.995	2.167	1.544	1.544	−1.19
TS-*tht*-1	1.926, 1.930	2.013	2.090, 2.500	1.970	1.551	−0.54
SO-O-*tht*	1.822, 1.839, 1.909	1.980	2.127, 2.193, 2.420	2.758	1.574	−1.46
TS-*tht*-2	1.832, 1.846, 1.864	1.923, 1.953	2.114, 2.138, 2.279	2.858	1.938	−1.29
S-O-O-*tht*	1.838, 1.843, 1.847	1.838, 1.843, 1.846	2.136, 2.137, 2.139	2.575	2.573	−1.69

**Table 4:** Bond lengths and energy change of the IS, TS, and FS for the dissociation of SO_2_-*ttt* on Ni(111) surface.

Configuration	Ni-O_a_	Ni-O_b_	Ni-S	S-O_a_	S-O_b_	ΔE_r_
SO_2_-*ttt*	2.019	2.038	2.129	1.554	1.559	−1.03
TS-1-*ttt*	1.925, 1.928	2.015	2.088, 2.469	1.969	1.555	−0.53
SO-O-*ttt*	1.822, 1.839, 1.907	1.979	2.125, 2.193, 2.426	2.747	1.575	−1.44
TS-2-*ttt*	1.835, 1.846, 1.865	1.924, 1.965	2.119, 2.147, 2.277	2.863	1.935	−1.29
S-O-O-*ttt*	1.838, 1.843, 1.847	1.838, 1.843, 1.846	2.136, 2.137, 2.139	2.575	2.573	−1.69

**Table 5:** The adsorption energy of SO_2_ on different metal-doped Ni(111) surfaces.

Types	Doping Metal				
	Pristine	Cu	Co	Rh	Pd
SO_2_-*tht*-1	−1.19	−0.77	−1.21	−0.87	−0.78
SO_2_-*tht*-2		−1.01	−1.07	−0.97	−0.94
SO_2_-*ttt*-1	−1.03	−0.73	−1.05	−0.64	−0.68
SO_2_-*ttt*-2		−0.50	−1.08	−0.76	−0.60
SO_2_-*ttt*-3		−1.09	−1.04	−0.64	−0.68
SO_2_-*nbt*-1	−1.23	−0.83	−1.18	−0.94	−0.83
SO_2_-*nbt*-2		−1.07	−1.28	−0.96	−0.88

**Table 6:** The adsorption energy (ΔE_ads_), reaction barriers (E_a_), and reaction energies (ΔE_r_) of SO_2_*-tht*-2 on various surfaces.

Reaction	Energy	Doping Metal				
		Pristine	Cu	Co	Rh	Pd
SO_2(g)_ → SO_2(ad)_	ΔE_ads_/eV	−1.19	−1.01	−1.07	−0.97	−0.94
SO_2(ad)_ → SO_(ad)_ + O_(ad)_	E_a_/eV	0.66	0.66	0.63	1.08	1.16
	ΔE_r_/eV	−0.27	0.00	−0.35	0.14	0.34
SO_(ad)_ + O_(ad)_ → S_(ad)_ + O_(ad)_ + O_(ad)_	E_a_/eV	0.17	0.39	0.06	0.69	0.60
	ΔE_r_/eV	−0.23	0.20	−0.33	0.50	0.09

## Data Availability

Data will be made available on request.

## Supplementary Materials

The following supporting information can be downloaded at https://www.mdpi.com/article/10.3390/molecules28186739/s1.
