# Supplementary material for: Decomposition of SO2 on Ni(111) Surface and the Effect of Metal Doping: A First-Principles Study
Source: Molecules. 2023 Sep 21;28(18):6739. doi: 10.3390/molecules28186739 (PMC10536407; doi:10.3390/molecules28186739)
Supplement: Supplementary file 1 [file molecules-28-06739-s001.zip › molecules-2591644-supplementary.pdf]

Supplementary Information

Decomposition of SO_2 on Ni(111) surface and the effect of metal doping: a first-principles study

Lingtao Liu, Chenxin Zhang, Wenshou Wang, Genghong Li, Bingtian Zhu

1. O and S adsorbed on different surfaces

Ni(111):

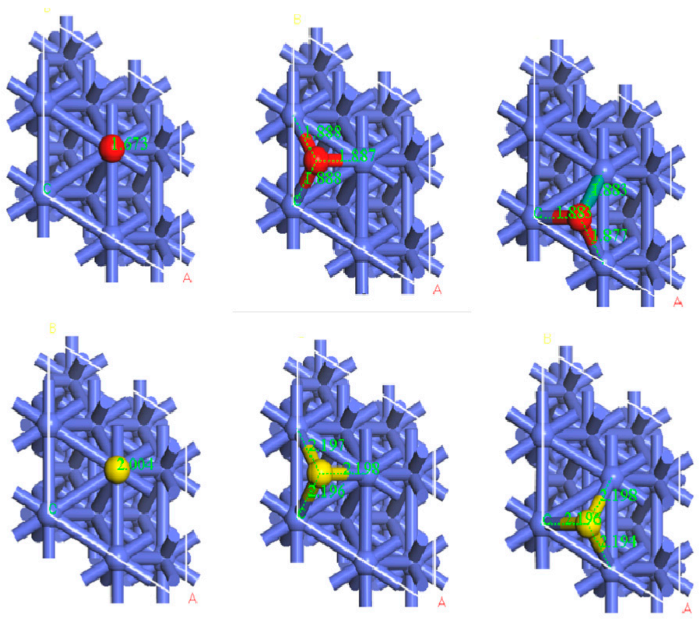

Cu(111):

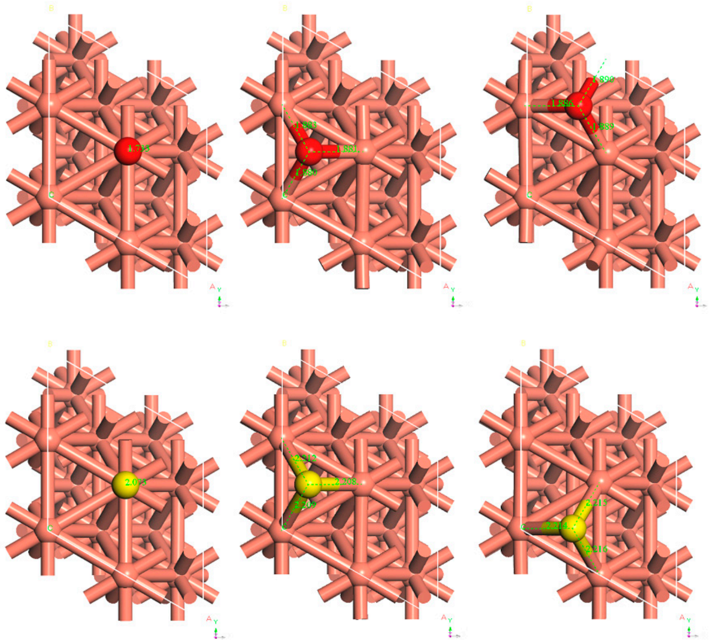

Rh(111):

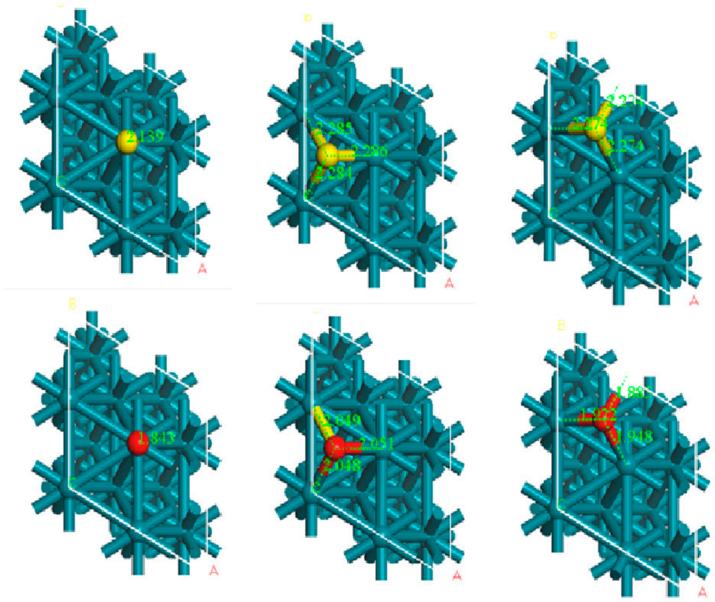

Pd(111):

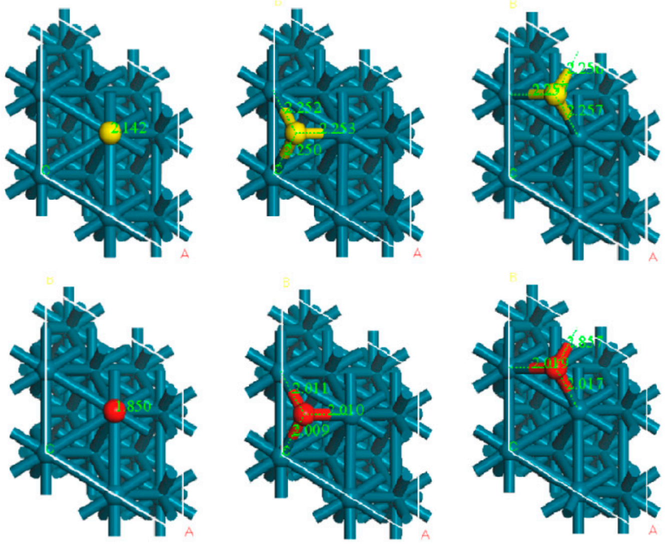

Fig. S1. Configurations and bond lengths of O and S adsorbed on different surfaces (The red and yellow spheres represent O and S atoms, respectively.)

2. SO_2 on pristine Ni(111)

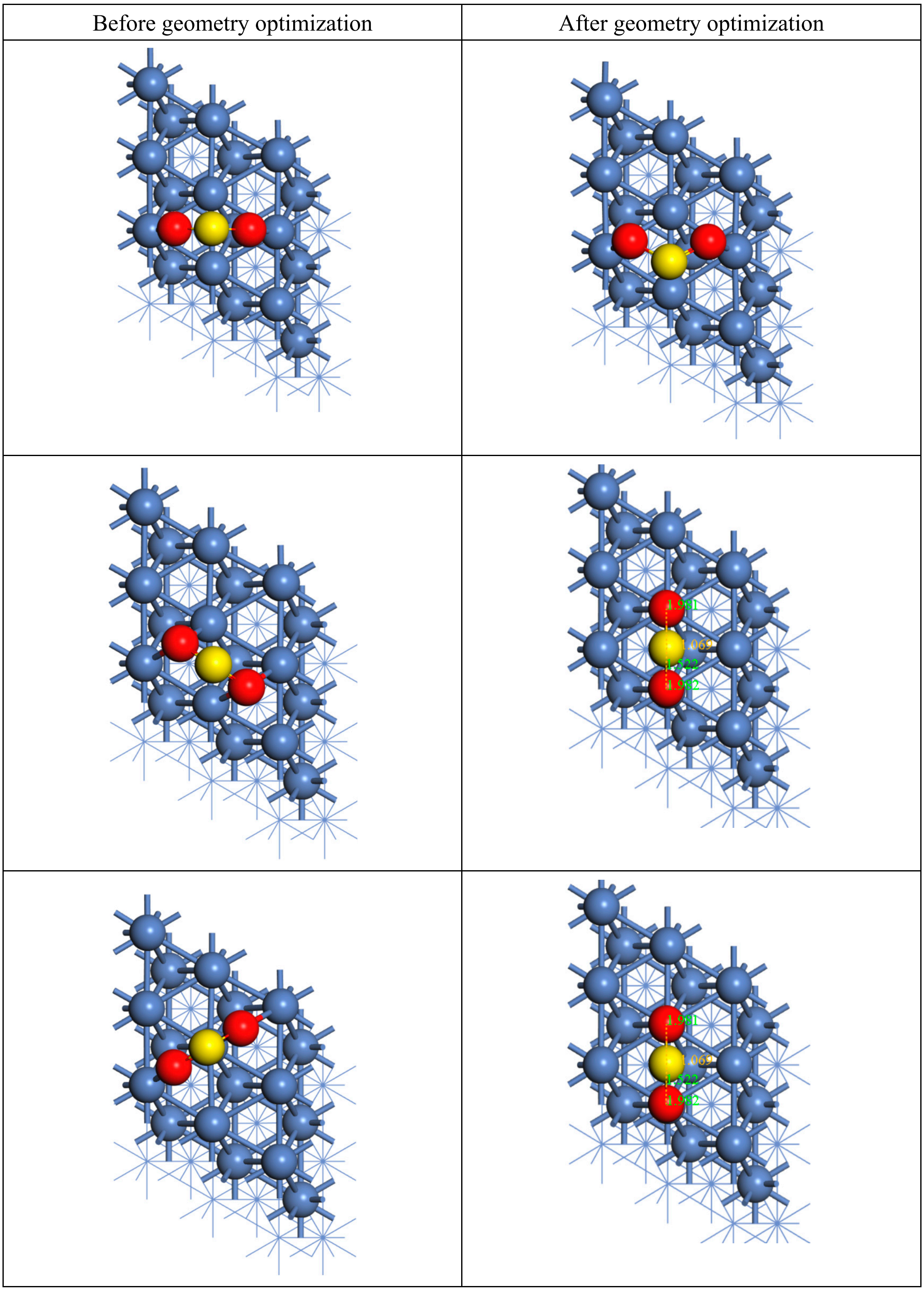

Fig. S2. Unstable configurations and the optimization results of SO_2 on pristine Ni(111)

3. SO_2 adsorbed on various doped surfaces

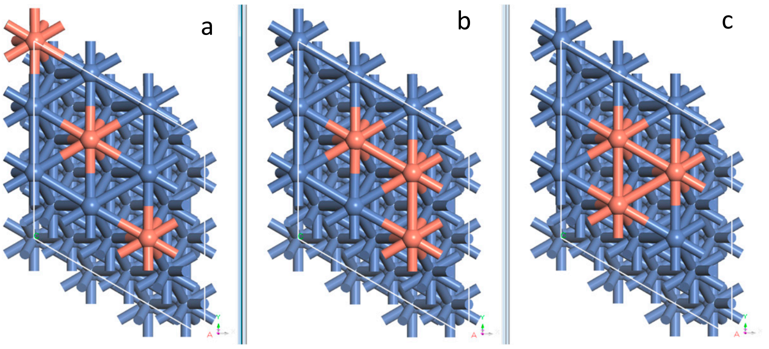

Fig. S3. Surface doping topologies(chose Cu-doping as an example)

Table S1. Energy difference of doped surfaces

eV	Co	Cu	Rh	Pd
a	base	base	base	base
b	+0.02	-0.03	+0.22	+0.06
c	+0.01	-0.06	+0.30	+0.18

Table S2. Bond length and bond angle of SO_2 on various doped surfaces

Doping metal	types	ΔH_a	S	Oa	Ob	S-Oa，S-Ob	∠O-S-O
Cu	1	-0.77	Cu-2.318	Ni-2.009	Ni-2.011	1.532,1.532	110.881
	2	-1.01	Ni-2.172	Ni-1.982	Cu-2.121	1.528,1.548	109.525
	3	-0.73	Ni-2.154	Cu-2.117	Cu-2.142	1.526,1.533	110.568
	4	-0.50	Cu-2.525	Ni-2.029	Ni-2.059	1.548,1.555	111.513
	5	-1.09	Ni-2.131	Ni-2.031	Ni-2.033	1.558,1.557	110.995
	6	-1.07	Ni-2.102<br>Cu-2.312	Ni-2.091	NM	1.519,1.458	114.558
	7	-0.83	Ni-2.157<br>Ni-2.160	Cu-2.196	NM	1.507,1.62	114.824
Co	1	-1.21	Ni-2.165	Ni-1.986	Co-2.024	1.547,1.547	108.554
	2	-1.07	Co-2.209	Ni-1.986	Ni-1.986	1.548,1.549	108.250
	3	-1.05	Ni-2.129	Ni-2.017	Ni-2.033	1.555,1.568	109.675
	4	-1.08	Ni-2.121	Co-2.061	Co-2.069	1.559,1.562	109.233
	5	-1.03	Co-2.151	Ni-2.066,2.105	Ni-2.015	1.543,1.613	107.964
	6	-1.18	Ni-2.153<br>Co-2.167	Ni-2.042	NM	1.532,1.460	113.689
	7	-1.28	Ni-2.135<br>Ni-2.142	Co-2.109	NM	1.525,1.460	114.398
Rh	1	-0.87	Ni-2.165	Ni-2.069	Rh-2.269	1.520,1.571	109.295
	2	-0.97	Rh-2.299	Ni-2.005	Ni-2.006	1.543,1.544	107.812
	3	-0.64	Ni-2.143	Ni-2.040	Ni-2.041	1.550,1.554	111.718

	4	-0.76	Rh-2.266	Ni-2.020	Ni-2.034	1.553,1.553	112.569
	5	-0.64	Ni-2.143	Ni-2.040	Ni-2.041	1.551,1.555	111.549
	6	-0.94	Ni-2.152,2.155	Ni-2.382	NM	1.502,1.463	115.088
	7	-0.96	Ni-2.149<br>Rh-2.284	Ni-2.095	Ni-2.095	1.524,1.456	114.374
Pd	1	-0.78	Ni-2.198	Ni-2.026	Pd-2.273	1.516,1.545	110.001
	2	-0.94	Pd-2.311	Ni-2.040	Ni-2.042	1.532,1.532	109.565
	3	-0.68	Ni-2.145	Ni-2.074	Ni-2.081	1.549,1.550	112.249
	4	-0.60	Pd-2.279	Ni-2.108	Ni-2.193	1.534,1.537	113.631
	5	-0.68	Ni-2.147	Ni-2.078	Ni-2.092	1.549,1.550	112.083
	6	-0.83	Ni-2.141<br>Rh-2.306	Ni-2.190	NM	1.455,1.509	115.687
	7	-0.88	Ni-2.166<br>Ni-2.169	Rh-2.369	NM	1.501,1.465	114.506

SO2-*tht*-1

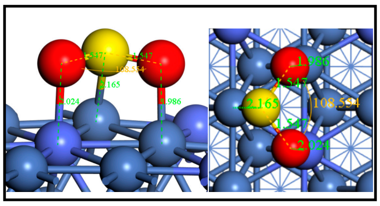

SO2-*tht*-2

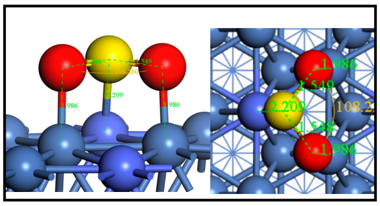

SO2-*ttt*-1

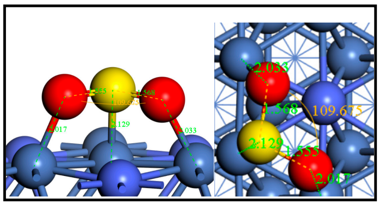

SO2-*ttt*-2

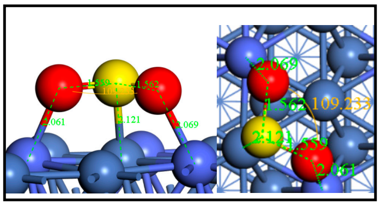

SO2-*ttt*-3

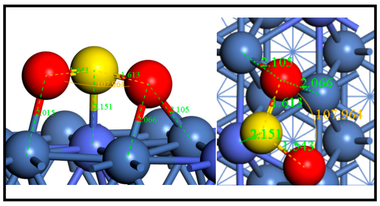

SO2-*nbt*-1

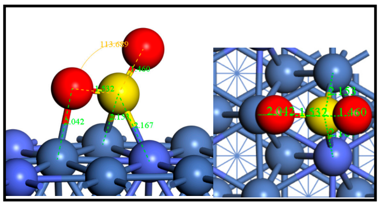

SO2-*nbt*-2

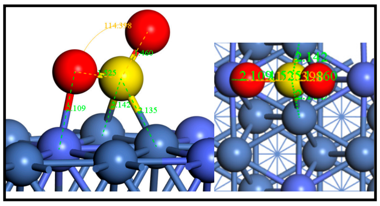

Fig. S4-1. Configurations of SO_2 on Co doped surfaces

SO2-*tht*-1

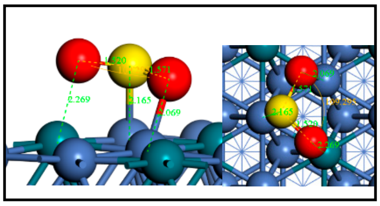

SO2-*tht*-2

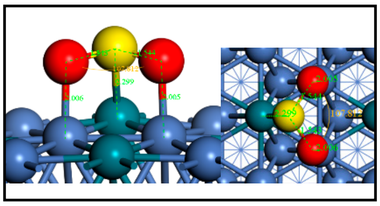

SO2-*ttt*-1

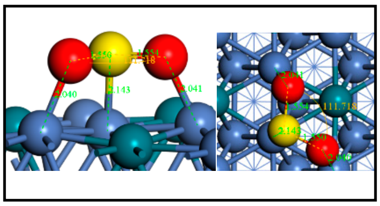

SO2-*ttt*-2

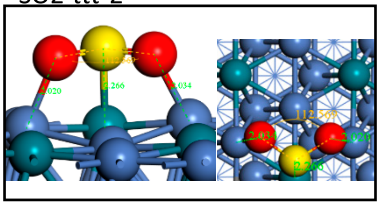

SO2-*ttt*-3

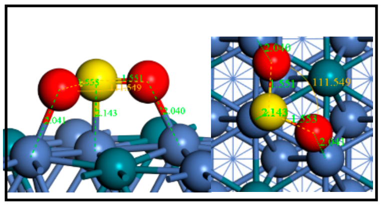

SO2-*nbt*-1

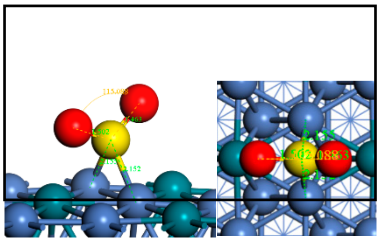

SO2-*nbt*-2

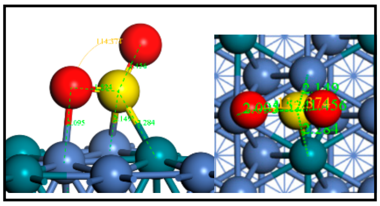

Fig. S4-2. Configurations of SO_2 on Rh doped surfaces

SO2-*tht*-1

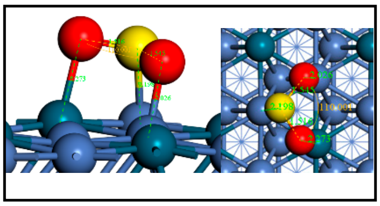

SO2-*tht*-2

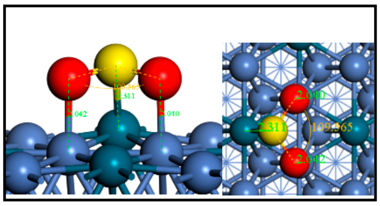

SO2-*ttt*-1

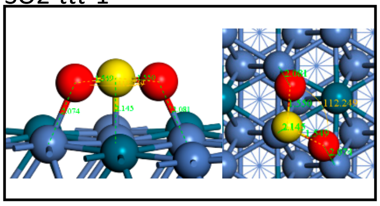

SO2-*ttt*-2

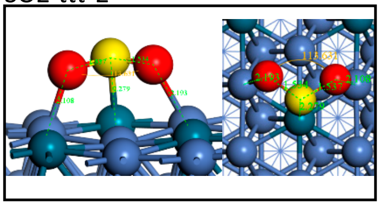

SO2-*ttt*-3

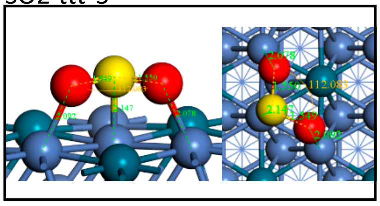

SO2-*nbt*-1

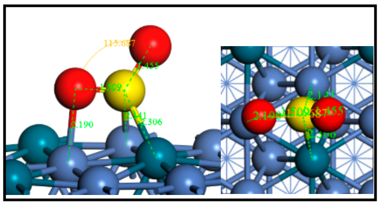

SO2-*nbt*-2

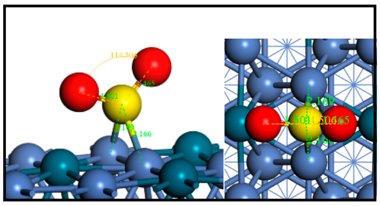

Fig. S4-3. Configurations of SO_2 on Pd doped surface

4. SO_2 decomposition on various doped surfaces

TS-1

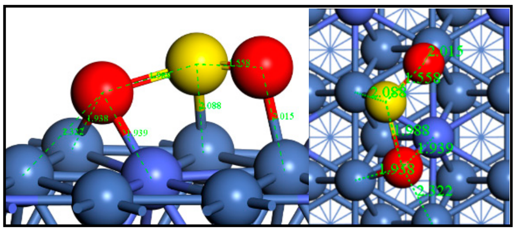

SO-O

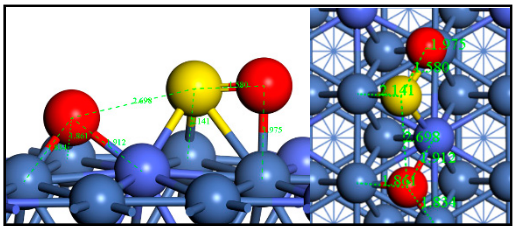

TS-2

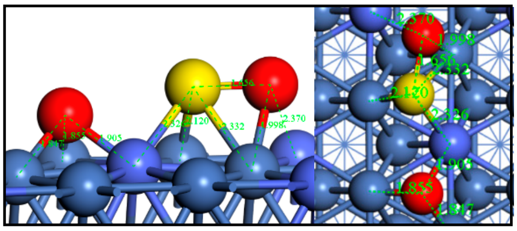

S-O-O

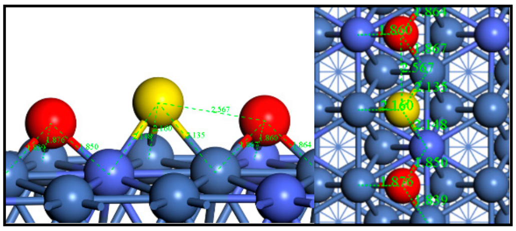

Fig. S5-1. Geometric structures of the dissociation of SO2-*tht*-2 on Co doped Ni(111) surface.(a, b: TS and FS of the first S-O dissociation; b, c, d): IS, TS, FS of the second S-O dissociation the IS.)

TS-1

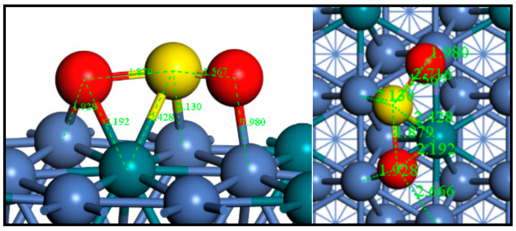

SO-O

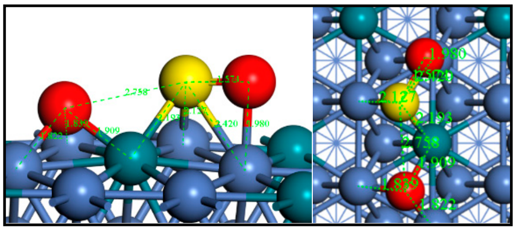

TS-2

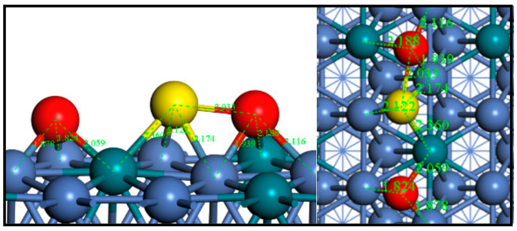

S-O-O

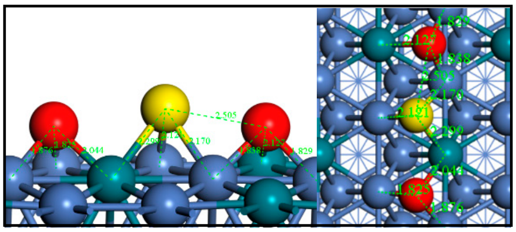

Fig. S5-2. Geometric structures of the dissociation of SO2-*tht*-2 on Rh doped Ni(111) surface.(a, b: TS and FS of the first S-O dissociation; b, c, d): IS, TS, FS of the second S-O dissociation the IS.)

TS-1

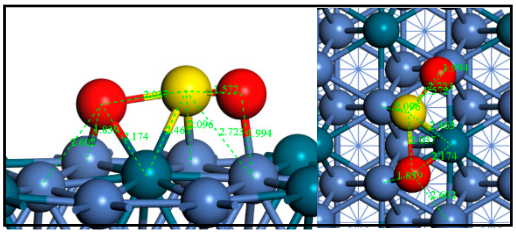

SO-O

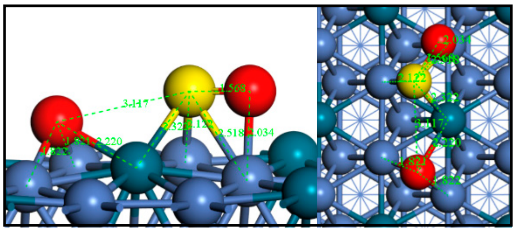

TS-2

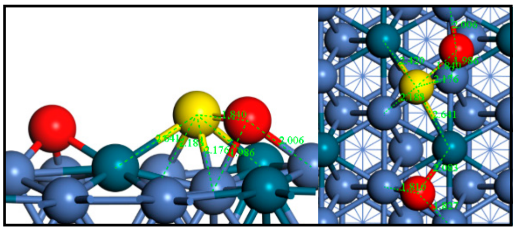

S-O-O

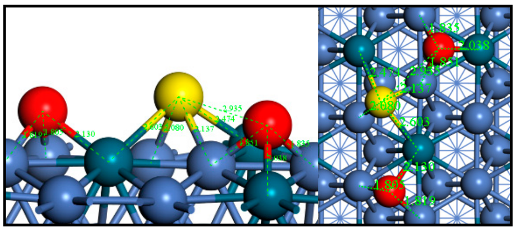

Fig. S5-3. Geometric structures of the dissociation of SO2-*tht*-2 on Pd doped Ni(111) surface.(a, b: TS and FS of the first S-O dissociation; b, c, d): IS, TS, FS of the second S-O dissociation the IS.)

5. PDOS of SO_2 adsorbed on various doped surfaces

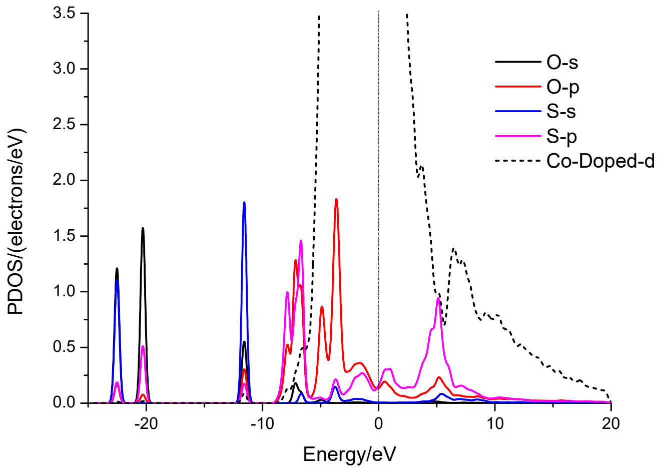

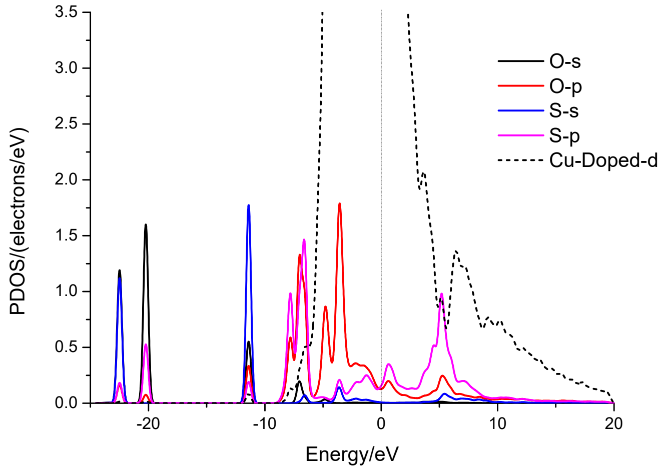

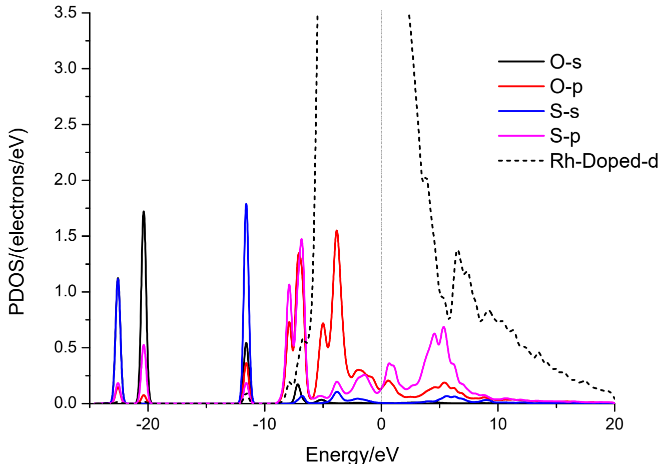

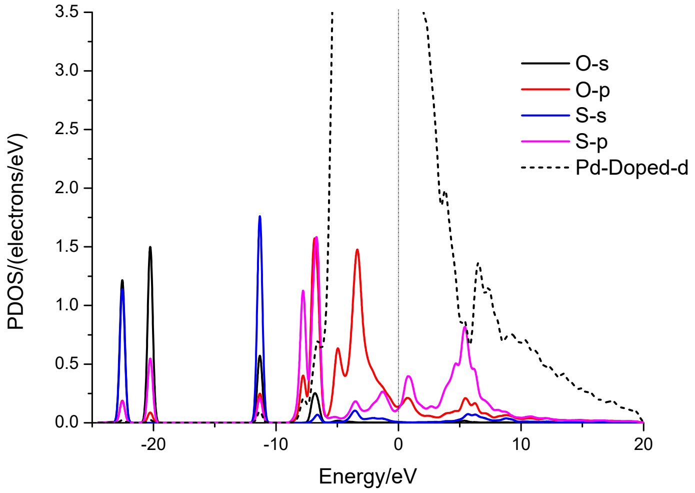

Fig. S6. PDOS of SO_2 adsorbed on various doped surfaces
